# A New Way to Wrap: Innovative ADM Braxon Packaging Technique Reduces Seroma in Prepectoral Breast Reconstruction

**DOI:** 10.3390/jcm15010021

**Published:** 2025-12-19

**Authors:** Giovanni Arrica, Corrado Rubino, Federico Ziani, Edoardo Filigheddu, Sofia De Riso, Gianluca Marcaccini, Roberto Cuomo, Claudia Trignano, Emilio Trignano

**Affiliations:** 1 Department of Medicine, Surgery and Pharmacy, University of Sassari, 07100 Sassari, Italy; corubino@uniss.it (C.R.); zianifederico@gmail.com (F.Z.); edofili@gmail.com (E.F.); sofia.deriso@aouss.it (S.D.R.); etrignano@uniss.it (E.T.); 2 Plastic Surgery Unit, University Hospital Trust of Sassari, 07100 Sassari, Italy; 3 Plastic and Reconstructive Surgery, Department of Medicine, Surgery and Neuroscience, University of Siena, 53100 Siena, Italy; gianlu32@gmail.com (G.M.); robertocuomo@outlook.com (R.C.); 4 Department of Biomedical Sciences, University of Sassari, 07100 Sassari, Italy; claudia.trignano@uniss.it

**Keywords:** prepectoral breast reconstruction, acellular dermal matrix (ADM), seroma prevention, surgical complications, level of evidence IV

## Abstract

**Background**: In recent years, prepectoral implant-based breast reconstruction supported by acellular dermal matrices (ADMs) has become an increasingly adopted alternative to submuscular techniques. Although this approach can improve patient comfort and aesthetic outcomes, fluid accumulation around the implant remains one of the most frequent and clinically relevant complications. Our unit progressively modified the handling and wrapping of the Braxon^®^ ADM with the aim of optimizing pocket configuration and reducing seroma formation. **Methods**: We performed a retrospective analysis of consecutive patients who underwent immediate prepectoral, direct-to-implant breast reconstruction with Braxon^®^ ADM at our institution between October 2019 and January 2025. Two techniques were compared: a standard full-wrap configuration and a modified approach in which the posterior ADM sheet is trimmed and only anterior coverage is maintained, with fenestrations created in the anterior wall. Clinical data and postoperative complications, including seroma rates, were recorded and compared between groups. Categorical variables were summarized as counts and percentages, and continuous variables as means and standard deviations. The primary comparison between techniques was performed on the presence of at least one postoperative complication using Fisher’s exact test, and odds ratios (OR) with 95% confidence intervals (CI) were calculated where appropriate. A *p*-value <0.05 was considered statistically significant. **Results**: A total of 138 direct-to-implant prepectoral reconstructions were included, 90 performed with the standard full-wrap technique and 48 with the modified wrapping approach. The overall complication rate was 27.8% (25/90) in the standard group and 10.4% (5/48) in the new-technique group. Seroma occurred in 8 patients (8.9%) in the standard group and in 1 patient (2.1%) in the modified-technique group. Fisher’s exact test demonstrated a significantly lower overall complication rate in the modified-technique cohort (10.4% vs. 27.8%; *p* = 0.02; OR 3.31; 95% CI 1.18–9.31), indicating that patients treated with the standard technique had approximately 3.3-fold higher odds of developing at least one complication than those treated with the modified technique. **Conclusions**: Anterior-only ADM coverage with selective trimming of the posterior sheet and fenestration of the anterior wall appears to reduce complications, particularly seroma, in ADM-assisted prepectoral breast reconstruction. Small technical refinements in ADM handling and pocket configuration, combined with a structured drainage protocol, may substantially improve postoperative outcomes in this setting.

## 1. Introduction

Alloplastic breast reconstruction techniques have continually evolved, with significant advances in terms of both aesthetic outcomes and patient safety [1,2].

The early clinical adoption of silicone breast implants in reconstructive surgery can be traced back to the pioneering work of Snyderman and Guthrie in 1971 [3], who placed the implants above the pectoral muscle rather than using the traditional submuscular approach [4,5]. This preserved the anatomical integrity of the pectoralis major. This technique later fell into disuse due to a high incidence of complications, including prosthetic exposure, capsular contracture, and implant dislocation [6].

Implant-based breast reconstruction has undergone substantial refinement over the last decade. The reintroduction of prepectoral implant positioning, together with the increasing availability of acellular dermal matrices (ADMs), has reshaped reconstructive strategies and expanded the indications for immediate single-stage procedures. Nevertheless, ADM-assisted prepectoral reconstruction remains associated with a spectrum of postoperative events, among which seroma formation is particularly frequent and can significantly influence both early recovery and long-term aesthetic outcomes.

After decades during which breast reconstruction was predominantly performed using a subpectoral approach, the continuous progress in both oncologic and reconstructive surgery, particularly in skin-sparing and nipple-sparing mastectomy techniques, has led to a renewed interest in the prepectoral plane. In this context, the development and availability of acellular dermal matrices (ADMs) have played a pivotal role by enabling complete implant coverage without the need to elevate the pectoralis major muscle.

Through the development of these new methods, prepectoral reconstruction with ADM has become one of the most widely adopted techniques for implant-based breast reconstruction in many centers, offering safety, effectiveness, and satisfactory aesthetic outcomes [7]. Braxon^®^ acellular dermal matrix (DECO Med S.r.l., Venice, Italy), a porcine collagen-based ADM, produced from decellularized porcine dermis and specifically designed for breast reconstruction [8], possesses unique tissue-specific properties, including resistance to immune rejection, reduction in dead space, improved weight distribution, and a decreased risk of skin flap necrosis [9].

Proper fixation of the ADM plays a crucial role in maintaining implant stability and ensuring intimate contact with the surrounding tissues, an essential prerequisite for revascularization, recellularization, and long-term integration.

This study describes the evolution of the prosthetic implant packaging technique using Braxon and evaluates the associated complication rates, with a specific focus on seroma formation. The modified wrapping technique was formally implemented in our unit after an internal audit conducted in late 2022 revealed that seroma remained the most frequent complication despite the adoption of standard ADM wrapping.

## 2. Materials and Methods

This study included all patients who underwent immediate prepectoral breast reconstruction using Braxon acellular dermal matrix (ADM) at the University Hospital Trust of Sassari, Italy, between October 2019 and January 2025.

The distribution of patients over the study period was as follows: 2 patients in 2019, 8 in 2020, 31 in 2021, 21 in 2022, 46 in 2023, 22 in 2024, and 8 in the first month of 2025.

All patients included in the analysis had a minimum postoperative follow-up of 6 months, ensuring complete assessment and documentation of all complications relevant to the study endpoints. Prior to surgery, all patients received comprehensive information regarding the prosthetic device, the nature of the surgical procedure, its indications, and the potential risks involved. Informed consent was obtained in writing. Standard preoperative assessments were performed in all cases, including routine laboratory blood tests and cardiologic evaluations, in accordance with national guidelines and institutional protocols [10].

All surgical procedures were carried out by the breast surgery team in collaboration with the plastic surgery unit. Mastectomies were performed by breast surgeons, while reconstructive procedures were carried out by plastic surgeons within a coordinated multidisciplinary setting.

Eligibility for immediate prepectoral reconstruction was restricted to patients presenting with tumors smaller than 5 cm, a tumor–muscle separation greater than 0.5 cm, no clinical or radiological signs of skin or chest wall invasion, absence of prior radiotherapy to the breast, and a body mass index lower than 35, adequate glycemic control, preservation of the subcutaneous tissue, and the presence of a well-perfused mastectomy skin flap. Patients were excluded from prepectoral reconstruction if they presented with tumors larger than 5 cm, a distance from the tumor to the pectoralis major muscle of less than 0.5 cm, involvement of the chest wall or axilla, locally advanced disease, or high risk of recurrence.

In the absence of exclusion criteria, all candidates underwent thorough preoperative evaluation to confirm their suitability for prepectoral placement. All procedures were performed in compliance with oncological safety standards, and patient selection was guided by strict criteria to ensure appropriate cancer control. Preoperative measurements and surgical planning were conducted on the day prior to surgery, including marking of anatomical landmarks and evaluation of skin flap quality [10].

During intraoperative assessment, only skin flaps demonstrating adequate perfusion were deemed suitable for immediate prepectoral reconstruction. Flap viability was assessed through both clinical and instrumental means [11]. Clinically viable flaps were characterized by the presence of active bleeding at the incision margins and a subcutaneous tissue thickness of at least 0.6 cm. The pinch test was routinely employed, alongside imaging modalities such as intraoperative angiography, to dynamically evaluate skin thickness and perfusion [12,13,14].

Indocyanine green (ICG) angiography was routinely performed in all cases, regardless of the fixation technique, as part of the standard intraoperative evaluation of mastectomy flap viability.

Smoking was regarded as a relative contraindication to immediate prepectoral reconstruction because of its association with impaired tissue perfusion and higher rates of postoperative complications. All patients were instructed to abstain from cigarette smoking for at least 30 days before surgery. As biochemical verification is not routinely performed in our unit, smoking status was based on patient self-report during preoperative evaluation, and patients who disclosed active smoking were excluded or deferred.

Patients who underwent neoadjuvant chemotherapy (NACT) were not excluded from the study. Eligibility criteria—including the requirement of a neoplastic mass <5 cm—were assessed during the preoperative evaluation carried out immediately before surgery. Accordingly, in patients treated with NACT, tumor size was measured post-NACT using preoperative imaging, and not at the time of initial diagnosis.

Once flap viability had been confirmed, implant selection was finalized following a thorough evaluation of the surgical pocket. Sizers were routinely used to determine the most appropriate implant volume and dimensions. Proper congruence between the implant and the surrounding space was considered essential to minimize dead space and to reduce the likelihood of postoperative complications, including seroma formation, rippling, wrinkling, and ischemic changes in the overlying skin [15,16,17,18,19,20].

### Surgical Technique and Differences from the Previous Approach

After mastectomy was completed, a multimodal assessment was performed to evaluate flap thickness and viability. This included direct visual inspection, the pinch test, and indocyanine green (ICG) fluorescence angiography. Once adequate perfusion and tissue integrity were confirmed, the preparation phase for prosthetic implantation began.

The weight of the excised glandular tissue served as a key factor in determining the appropriate implant volume. It has been observed that implants exceeding 500–550 g are associated with a higher incidence of postoperative complications and less satisfactory aesthetic outcomes. In patients with an estimated preoperative breast volume greater than 500 g, contralateral breast reduction was recommended to avoid exceeding this threshold [21,22].

In selected cases, additional reshaping procedures such as lipofilling may also considered to optimize symmetry and projection [23,24].

Implant selection was guided by a combination of patient preference and anatomical parameters, including chest wall and breast dimensions (width, height, and glandular projection). Trial implants (sizers) were used to identify the optimal prosthesis, which was then positioned within the prepared pocket while the patient was placed in a semi-sitting position to optimize symmetry with the contralateral breast.

Once the final implant had been selected, all subsequent handling of the acellular dermal matrix (ADM) was performed under rigorously sterile conditions to prevent any device-related contamination. A suction drain was inserted, and the prosthetic pocket was irrigated first with a saline and hydrogen peroxide solution, followed by antibiotic solution.

**Figure 1:**
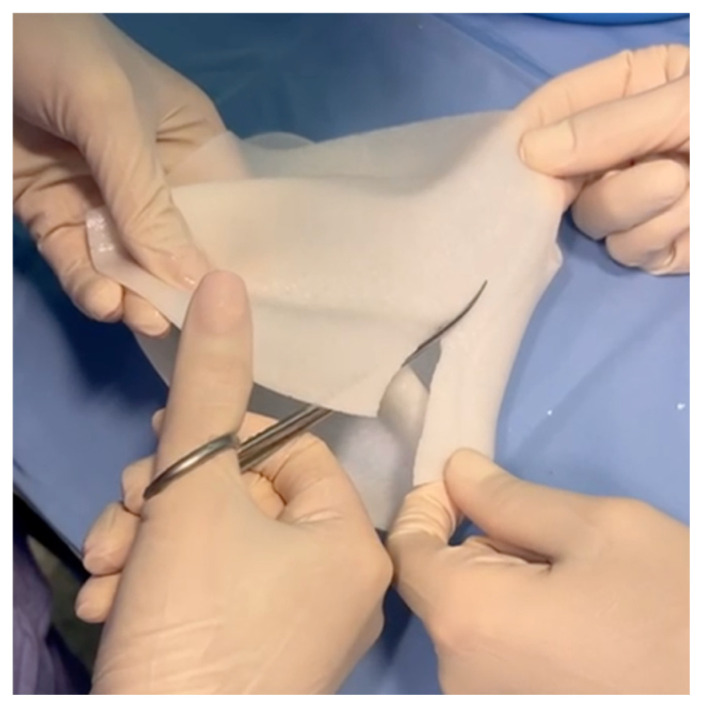
Excision and removal of the cranial portion of the posterior wall of the Braxon^®^ ADM during the trimming process.

**Figure 2:**
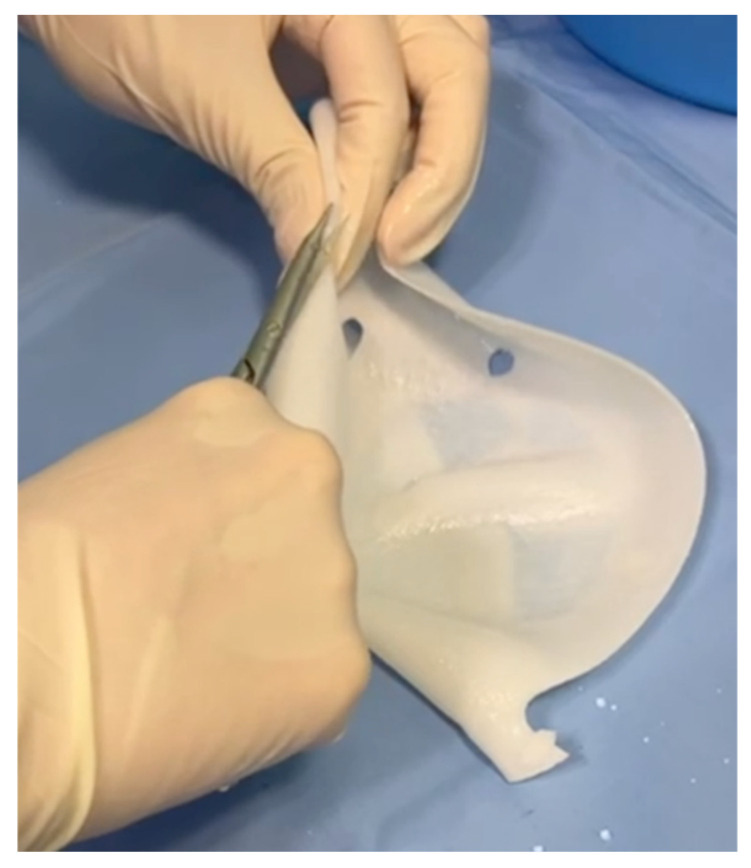
Creation of fenestrations in the anterior wall to facilitate postoperative fluid drainage.

**Figure 3:**
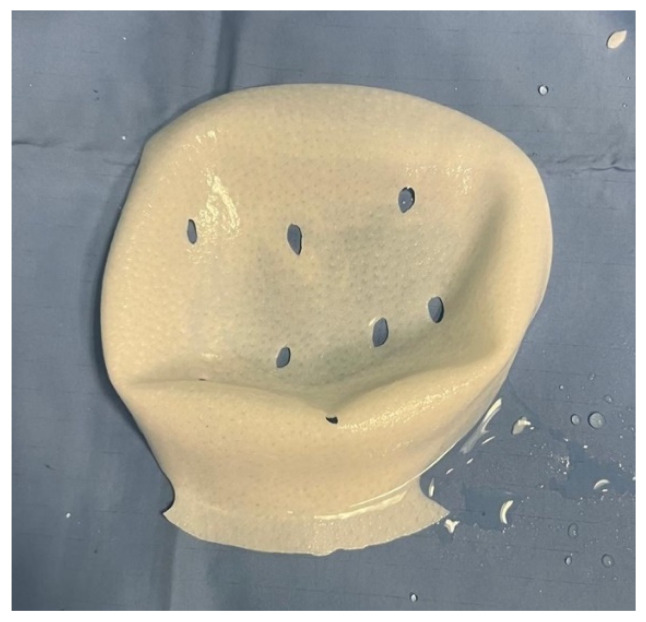
Final appearance of the ADM after posterior trimming and anterior fenestration.

In the standard technique [25], a pre-shaped Braxon^®^ acellular dermal matrix was used to perform a full-wrap reconstruction. The implant was completely enveloped by the ADM on the back table before insertion into the prepectoral pocket, creating 360-degree coverage around the device. In the new technique, the ADM provides anterior coverage only. The matrix is placed over the anterior surface of the implant and fixed directly to the pectoral fascia with multiple sutures, without forming a complete envelope around the prosthesis. The anterior sheet is fenestrated to facilitate fluid passage and reduce dead space between the implant and the overlying mastectomy flap. Specifically, the cranial portion of the posterior wall is removed (Figure 1), and fenestrations are created in the anterior wall to facilitate fluid drainage (Figure 2 and Figure 3).

To ensure reproducibility, the estimated 40% reduction in ADM surface area was calculated intraoperatively by visually comparing the trimmed ADM template with the original full-wrap Braxon^®^ membrane. The cranial resection of the posterior wall and the fenestrations applied to the anterior sheet were outlined and proportionally compared with the untouched membrane surface, allowing a simple geometric estimation of the residual usable area.

**Figure 4:**
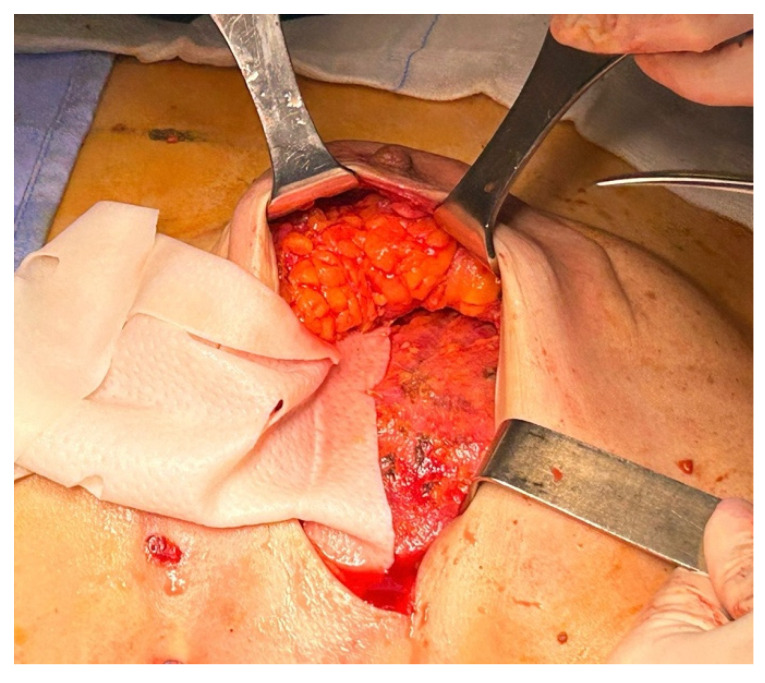
Anchorage of the ADM to the pectoralis major fascia using interrupted absorbable sutures.

**Figure 5:**
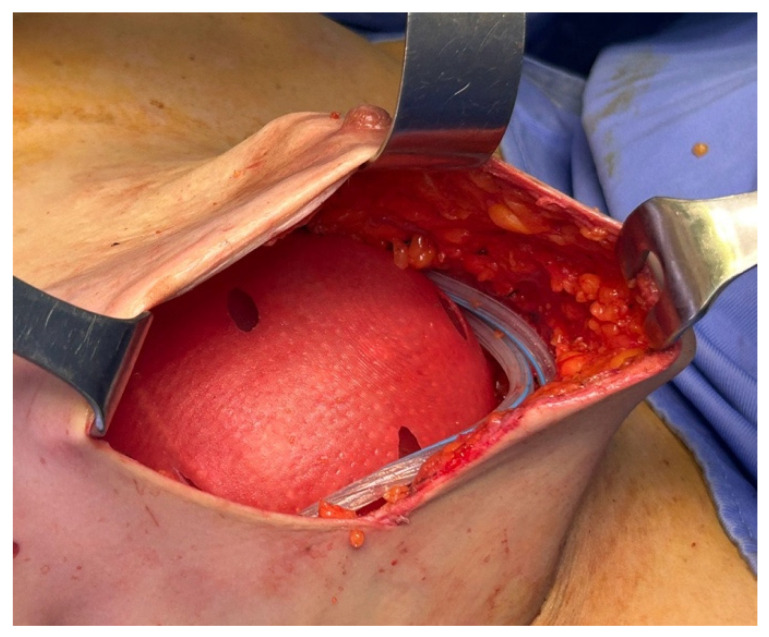
Coverage of the anterior surface of the breast implant with the trimmed and fenestrated anterior portion of the ADM.

Once the membrane was inserted into the pocket, it was anchored to the pectoralis major fascia using interrupted Vicryl 2/0 sutures (Figure 4). The definitive implant was then placed, and the anterior surface of the prosthesis was covered by the anterior portion of the ADM (Figure 5). Both the membrane and the underlying implant were secured to the pectoralis major, the serratus anterior, and the adjacent muscular fascia using interrupted resorbable sutures (typically 12–15 Vicryl 2/0 stitches), spaced at least 1 cm apart.

In both techniques, two to three superficial sutures were placed between the ADM and the subcutaneous layer of the mastectomy flap to enhance anterior stability. These sutures did not transfix the mastectomy flap or involve the NAC and were identical in number and position in both the standard and the new technique. Postoperative instructions included the use of a surgical compression bra for a minimum of 30 days, to maintain implant positioning and ensure continuous adhesion between the ADM and host tissue, thus promoting optimal integration.

Arm mobility on the affected side was restricted for 7 to 15 days to reduce the risk of seroma formation and to support early recovery. Drain output was closely monitored in all patients. In the standard-technique group, drains were maintained for a minimum of 14 days independently of output, according to the previous institutional protocol. In the new-technique group, the drainage protocol was updated: drains were removed once output was <30 mL/24 h for two consecutive days, which typically occurred around postoperative day 12. This volume-based criterion replaced the fixed-duration approach and ensured more tailored postoperative fluid management. In most cases, patients were discharged with the drains still in place. During the first two postoperative weeks, patients received daily clinical evaluations, during which the general condition of the reconstruction was assessed and drainage output was recorded. Follow-up visits were scheduled at 1, 3, and 6 months postoperatively, with appropriate wound care and management of any complications that occurred.

Seroma was defined as a clinically evident fluid collection requiring clinical assessment (with or without aspiration). Small asymptomatic fluid collections detected incidentally on imaging and not requiring intervention were not classified as seroma.

## 3. Statistical Analysis

The primary outcome of the study was the occurrence of at least one postoperative complication. This composite endpoint was chosen to provide sufficient statistical power for comparison between techniques, given the limited number of events for individual complications. Seroma, as the most frequent and clinically relevant complication in ADM-assisted prepectoral reconstruction and the event most plausibly influenced by the modified ADM-handling technique, was treated as the main specific complication of interest and its incidence was described separately. Other postoperative complications (skin/nipple necrosis, infection, wound dehiscence, and implant loss) were also collected and reported descriptively; no formal hypothesis testing was planned for individual complications, as the study was not powered to detect differences for these separate outcomes. The association between surgical approach and postoperative complication rate was examined through comparative statistical testing. Patient distribution according to complication status in each group is reported in the contingency matrix shown in Table 1.

Among the patients treated with the standard technique, 65 out of 90 experienced no complications, whereas 25 encountered at least one postoperative event. In the group treated with the new technique, 43 out of 48 patients had no complications, while 5 experienced postoperative issues. Overall, the study included a total of 138 patients, of whom 108 had no complications and 30 developed one or more complications following surgery.

Percentages in Table 1 refer to the proportion of complications within each column, representing the distribution of all patients with postoperative complications between the two groups.

Categorical variables, including the presence of at least one postoperative complication, were compared between groups using Fisher’s exact test. This test was selected because of the low number of observed events, particularly in the new-technique group, which precluded the use of reliable multivariable regression models. Effect size was expressed as odds ratios (OR) with corresponding 95% confidence intervals (CI). All statistical tests were two-sided, and a *p*-value <0.05 was considered statistically significant. Statistical analyses were carried out using standard methods for categorical data. Due to the retrospective design of the study and the absence of a standardized classification system for the severity of postoperative complications, such as the Clavien–Dindo classification [26], complications were analyzed in aggregate rather than stratified by grade or clinical relevance.

## 4. Results

Between October 2019 and January 2025, 138 consecutive immediate prepectoral breast reconstructions assisted by acellular dermal matrix were carried out at our institution. The standard fixation technique was adopted in 90 cases, whereas 48 procedures were performed using the novel fixation approach. Patients had a mean age of 55.2 ± 11.6 years and a mean body mass index of 24.1 ± 3.9. Implant volumes showed a mean value of 317 mL, with a minimum of 150 mL and a maximum of 510 mL.

Prior to the adoption of the new technique, the most frequently observed complications were seroma, occurring in 8.9% of cases, followed by skin necrosis in 6.7%, implant loss in 4.4%, wound dehiscence in 4.4%, and infection in 3.3%. After the new wrapping and fixation method was implemented, a marked reduction in the overall complication rate was observed. In the group treated with the new technique, a total of five patients experienced complications. These included one case of seroma and one case of nipple necrosis, both of which resolved within 25 days, as well as one case each of implant loss, wound dehiscence, and infection. In the new-technique group, drains were removed once output was <30 mL/24 h (mean 12 days), whereas patients treated with the standard technique followed the previous institutional protocol with a minimum drain duration of 14 days. Table 2 summarizes all individual postoperative complications in both groups. Seroma, skin/nipple necrosis, wound dehiscence, infection, and implant loss were all less frequent in the new-technique group. Some patients experienced more than one complication; therefore, totals may exceed the number of patients with ≥1 event.

Fisher’s exact test demonstrated a statistically significant reduction in postoperative complications in patients treated with the new technique (5/48, 10.4%) compared with the standard technique (25/90, 27.8%) (*p* = 0.02; OR 3.31; 95% CI 1.18–9.31).

No multivariable regression analysis was performed, as the small number of events in the new-technique group did not allow for a stable model.

## 5. Discussion

Patients with a normal body mass index, limited comorbidities, and well-vascularized mastectomy flaps are generally considered ideal candidates for prepectoral breast reconstruction. Although neither a BMI up to 35 nor the presence of macromastia constitutes an absolute contraindication, both factors may increase the likelihood of postoperative complications and technical complexity. In patients with macromastia, a skin-reducing mastectomy technique is routinely adopted to optimize reconstructive outcomes [20].

Compared with the traditional two-stage expander/implant approach, immediate breast reconstruction performed at the time of mastectomy offers comparable safety and clinical outcomes, while achieving similar levels of patient satisfaction and health-related quality of life [27,28]. Among the most frequently cited benefits of this approach are improved aesthetic results and greater patient-reported satisfaction, making it a favorable option for appropriately selected cases [29]. The practice of immediate reconstruction using the prepectoral approach has gained widespread acceptance, as it ensures oncologic safety, is associated with low morbidity, and significantly enhances postoperative quality of life for many patients [30].

Despite these advantages, common complications associated with this reconstructive technique include seroma, soft tissue necrosis, and rippling. In a retrospective study by Chun YS et al. [31], the use of acellular dermal matrix (ADM) was associated with a more than fourfold increase in seroma risk compared to reconstructions without ADM, with a statistically significant *p*-value <0.05. In a separate analysis, Caputo et al. [32] reported an overall seroma rate of 4.9% in cases using the prosthetic wrapping technique with Braxon, although they concluded that the ADM itself was not a direct causal factor.

All postoperative complications were systematically collected and reported for transparency. From a clinical standpoint, seroma was the most relevant complication of interest, but given the limited number of events for individual outcomes, the formal statistical comparison between techniques was performed on the composite endpoint of “any postoperative complication”, whereas specific complications (including seroma) were analyzed descriptively. The reduction in skin and nipple necrosis observed with the new technique cannot be attributed to differences in ICG use, as intraoperative angiography was applied uniformly in both groups. The improvement is mainly related to the technical modifications introduced, particularly the anterior ADM fenestrations, which facilitate fluid passage and reduce the accumulation of serous collections between the implant and the matrix. This enhances ADM–tissue contact, decreases local pressure and shear forces, limits dead space, and thereby improves flap perfusion.

The primary objective of the modified wrapping technique was to reduce dead space and fluid accumulation at the implant–ADM interface. Intraoperative geometric estimation, performed by comparing the trimmed ADM template with the full Braxon membrane, indicated that the portion of ADM effectively excluded from use corresponds to approximately 40% of the original surface area. This estimate derives from proportional assessment of the resected posterior wall and the fenestrated anterior portion. Specifically, about 35% of the posterior wall is excised, while an additional 5% is eliminated through the creation of fenestrations in the anterior wall. The rationale behind this surgical approach is that the anterior wall of the ADM, which is critical for prosthesis stabilization, is preserved in nearly its entirety. The lower portion of the posterior wall is also maintained to support the implant’s position and prevent downward displacement. Fenestrations in the anterior wall are designed to facilitate drainage of fluid from the implant–ADM interface, thereby minimizing potential seroma accumulation.

The omission of posterior ADM coverage in the new technique provides several mechanical and biological advantages compared with the full-wrap configuration. The full envelope inherently increases ADM bulk and introduces redundant material behind the implant, which may create unnecessary tension or exert pressure on the mastectomy flap and surrounding tissues. By contrast, anterior-only coverage reduces the amount of ADM used and eliminates posterior dead space, thereby improving the apposition between the implant, the ADM, and the overlying flap.

The reduction in seroma observed with the new technique is mainly related to the modification of the implant–ADM interface. In the early postoperative phase, the acellular dermal matrix is not yet adherent to the surrounding tissues, and potential spaces may form where serous fluid tends to accumulate. By selectively reducing the posterior ADM coverage, the extent of these potential spaces is limited. In addition, the anterior fenestrations prevent the formation of a closed periprosthetic chamber, allowing fluid to drain toward the suction drains instead of remaining trapped between the implant and the matrix. When combined with our standardized drainage protocol, this configuration promotes continuous evacuation of postoperative fluid and provides a plausible mechanical explanation for the lower seroma rate observed with the modified technique. In continuity with our previous technique, we routinely place two suction drains, consistent with the practice described by George et al. [33]. Onesti et al., by contrast, utilized a single vacuum drain placed in the inframammary fold, which was removed once the daily drainage volume dropped below 30 mL, a protocol partially aligned with ours [34]. However, with the introduction of the modified technique, the postoperative drainage strategy was slightly simplified, and drains were removed earlier, at approximately 12 days instead of the previous 14-day protocol. Therefore, the observed reduction in seroma rates is most plausibly explained by the combined effect of the modified ADM configuration—with reduced posterior coverage and anterior fenestrations—and the structured postoperative management adopted in our unit. In particular, the smaller ADM surface and the absence of a closed periprosthetic chamber limit potential fluid spaces, while the drainage policy, external compression, intraoperative antibiotic irrigation and temporary limitation of shoulder movement help promote continuous evacuation of postoperative fluid. Urquia et al. reported that infection represented the most frequent cause of surgical reintervention, affecting 7.65% of reconstructed breasts, thereby underscoring the relevance of our finding of a very low infection rate (2.1%) observed in the cohort treated with our refined surgical technique [35]. The combination of an adequately maintained drainage period, intraoperative antibiotic irrigation, consistent external compression, and temporary restriction of shoulder mobility may have contributed to the observed reduction in seroma formation, with a measured incidence of only 2.08%.

Hematoma, another potential complication in breast reconstruction, was not observed in our cohort. This finding contrasts with reported rates as high as 23% in conventional approaches, in line with previous studies focusing on direct-to-implant reconstructions [36]. Within the limits of this retrospective study, the reduction in postoperative complications seems largely linked to the specific technical changes introduced in ADM handling and implant fixation. Due to the limited number of events, the influence of patient-related variables such as age and BMI on early postoperative outcomes could not be reliably assessed.

While our data indicate a clear reduction in overall postoperative complications, largely attributable to the technical standardization of implant positioning and ADM management, it remains essential to acknowledge and address residual risks to achieve durable clinical and aesthetic results. The incorporation of scheduled follow-up visits and targeted biomodelling procedures has become a standard component of our workflow, particularly for patients pursuing further refinement of cosmetic outcomes after initial reconstruction [37].

This study is inherently limited by its retrospective design. Moreover, the duration of follow-up did not allow for long-term evaluation of either oncologic safety or final aesthetic outcomes, which warrants further prospective investigation.

A technique aimed at improving upper pole fullness was proposed by Cuomo et al., consisting of a glandular-sparing mastectomy in which approximately 1 cm of residual glandular tissue and about 2 cm of subcutaneous fat are preserved in the superior pole. When applied to selected cases, this approach has been associated with encouraging aesthetic outcomes [38]. In cases presenting with grade 2–3 rippling or wrinkling, we have achieved successful correction through autologous fat grafting (lipofilling), confirming the clinical value of secondary contouring procedures. Although oncologic safety remains our institutional priority—typically involving preservation of less than 2 cm of anterior dermal tissue—selected aesthetic approaches such as those proposed by Cuomo et al. provide meaningful guidance in patients with high cosmetic expectations [38].

Vidya et al. proposed a modification of ADM handling involving posterior fenestrations to promote seroma drainage and minimize fluid accumulation. In contrast, our technique is based on fenestration of the anterior wall and selective resection of the upper portion of the posterior wall of the matrix [39].

Cattelani et al. demonstrated that prepectoral reconstructions using acellular dermal matrix (ADM) are associated with reduced postoperative pain and improved upper limb function when compared to subpectoral or expander-based methods, supporting the rationale behind our preference for muscle-sparing protocols [40]. As ADM-assisted prepectoral reconstruction continues to gain widespread acceptance in clinical practice, many breast centers are now adopting this approach as a first-line option for appropriately selected patients [40,41,42].

It is important to acknowledge that the observed reduction in complication rates may, in part, reflect a progressive improvement in surgical expertise and familiarity with ADM handling over time. Although these results are encouraging, they must be interpreted within the specific clinical and institutional context of the present study. Broader validation in prospective or multicenter settings would be necessary to confirm the reproducibility and generalizability of these findings.

Despite the ongoing concern regarding the cost of ADM, the clinical advantages achieved through our standardized and reproducible technique support its broader adoption in centers aiming to reduce postoperative complications based on measurable, evidence-based outcomes. In our experience, the reduction in complication rates associated with the new technique translated into a lower frequency of reinterventions and improved early patient satisfaction. Although these benefits were observed within the scope of a specific surgical setting, they may contribute to reducing the overall postoperative workload and optimizing the use of healthcare resources in similar clinical environments.

## 6. Conclusions

The refinement of the ADM Braxon wrapping technique in prepectoral breast reconstruction has been associated with a reduction in seroma rates, which remain the most frequent complication in this surgical context, when compared to the previously adopted standard method.

Our findings support the effectiveness of this modified approach, emphasizing, within a standardized postoperative protocol including compression and controlled drainage, the central role of intentional ADM surface area reduction achieved through selective posterior trimming and controlled anterior fenestrations, which was estimated intraoperatively to result in a substantially smaller ADM footprint.

In our clinical experience, even minor technical adjustments, when applied systematically, can result in measurable decreases in complication rates and contribute to improved short-term patient outcomes. Overall, these results indicate that carefully standardizing ADM handling and implant fixation can help reduce complications and make ADM-assisted prepectoral reconstruction safer in everyday practice.

## Figures and Tables

**Table 1:** Comparison of postoperative complication rates according to surgical technique.

Technique	No. Patients	No Complications	Complications
Old	90	65	25
	(65.2%)	(60.2%)	(83.3%)
New	48	43	5
	(34.8%)	(39.8%)	(16.7%)
Total	138	108	30
	(100%)	(100%)	(100%)

Percentages calculated per column. *p*-value calculated with Fisher’s exact test = 0.02.

**Table 2:** Distribution of specific complications according to surgical technique.

Complications	Old Technique (*n* = 90)	New Technique (*n* = 48)
Seroma	8 (8.9%)	1 (2.1%)
Skin/Nipple Necrosis	6 (6.7%)	1 (2.1%)
Implant Loss	4 (4.4%)	1 (2.1%)
Wound Dehiscence	4 (4.4%)	1 (2.1%)
Infection	3 (3.3%)	1 (2.1%)
Patient with ≥1 Postoperative Complication	25 (27.8%)	5 (10.4%)

Some patients experienced more than one complication; therefore, the sum of individual categories exceeds the number of patients with at least one complication.

## Data Availability

The data used or analyzed during the current study are available from the corresponding author upon reasonable request.
